# Frequency and characterization of the use of cuffed tracheal tubes in neonatal and pediatric intensive care units in Brazil

**DOI:** 10.5935/0103-507X.20200038

**Published:** 2020

**Authors:** João Paulo Berti Buzzi Rodrigues, Suzi Laine Longo dos Santos Bacci, Janser Moura Pereira, Cíntia Johnston, Vivian Mara Gonçalves de Oliveira Azevedo

**Affiliations:** 1 Programa de Residência em Área Profissional da Saúde (Uni e Multiprofissional), Faculdade de Medicina, Universidade Federal de Uberlândia - Uberlândia (MG), Brasil.; 2 Faculdade de Matemática, Universidade Federal de Uberlândia - Uberlândia (MG), Brasil.; 3 Departamento de Pediatria, Faculdade de Medicina, Universidade de São Paulo - São Paulo (SP), Brasil.; 4 Faculdade de Educação Física e Fisioterapia, Universidade Federal de Uberlândia - Uberlândia (MG), Brasil.

**Keywords:** Cannula, Trachea, Infant, Newborn, Intubation, intratracheal/instrumentation, Intensive care units, neonatal, Intensive care units, pediatric, Cânula, Traqueia, Lactente, Recém-nascido, Intubação intratraqueal/instrumentação, Unidades de terapia intensiva neonatal, Unidades de terapia intensiva pediátrica

## Abstract

**Objective:**

To identify the neonatal, pediatric and mixed (neonatal and pediatric) intensive care units in Brazil that use cuffed tracheal tubes in clinical practice and to describe the characteristics related to the use of protocols and monitoring.

**Methods:**

To identify the intensive care units in Brazil, the Ministry of Health’s National Registry of Health Facilities was accessed, and information was collected on 693 registered intensive care units. This was an analytical cross-sectional survey conducted through electronic questionnaires sent to 298 neonatal, pediatric and mixed intensive care units in Brazil.

**Results:**

This study analyzed 146 questionnaires (49.3% from neonatal intensive care units, 35.6% from pediatric intensive care units and 15.1% from mixed pediatric intensive care units). Most of the participating units (78/146) used cuffed tracheal tubes, with a predominance of use in pediatric intensive care units (52/78). Most of the units that used cuffed tracheal tubes applied a cuff pressure monitoring protocol (45/78). The use of cuff monitoring protocols was observed in intensive care units with a physical therapy service exclusive to the unit (38/61) and in those with a physical therapist present 24 hours/day (25/45). The most frequent cause of extubation failure related to the use of cuffed tracheal tubes in pediatric intensive care units was upper airway obstruction.

**Conclusion:**

In this survey, the use of cuffed tracheal tubes and the application of a cuff pressure monitoring protocol was predominant in pediatric intensive care units. The use of a monitoring protocol was more common in intensive care units that had a physical therapist who was exclusive to the unit and was present 24 hours/day.

## Abstract (pt)

**Objetivo:**

Identificar as unidades de terapia intensiva neonatais, pediátricas e mistas (neonatais e pediátricas) no Brasil que utilizam cânulas traqueais com balonete na prática clínica, e descrever as características relacionadas à utilização de protocolos e monitoração.

**Métodos:**

Para identificação das unidades de terapia intensiva no Brasil, foi acessado o Cadastro Nacional de Estabelecimentos de Saúde do Ministério da Saúde, e foram obtidas informações de 693 unidades de terapia intensiva cadastradas. Trata-se de estudo transversal analítico do tipo *survey* realizado por questionário eletrônico enviado para 298 unidades de terapia intensiva neonatais, pediátricas e mistas do Brasil.

**Resultados:**

Este estudo analisou 146 questionários (49,3% de unidades de terapia intensiva neonatais, 35,6% de unidades de terapia intensiva pediátricas e 15,1% de unidades de terapia intensiva pediátricas mistas). A maioria das unidades participantes (78/146) utilizou cânulas traqueais com balonete, com predomínio de uso nas unidades de terapia intensiva pediátricas (52/78). A maioria das unidades que utilizou cânulas traqueais com balonete aplicou protocolo de monitoração da pressão do balonete (45/78). O uso de protocolos de monitoração do balonete foi observado nas unidades de terapia intensiva com Serviço de Fisioterapia exclusivo da unidade (38/61) e naquelas com tempo de atuação do fisioterapeuta 24 horas/dia (25/45). A causa de falha de extubação mais frequentemente relacionada ao uso de cânulas traqueais com balonete em unidades de terapia intensiva pediátricas foi a obstrução de vias aéreas superiores.

**Conclusão:**

Nesta enquete, houve predomínio do uso de cânulas traqueais com balonete e da aplicação de protocolo de monitoração da pressão do balonete em unidades de terapia intensiva pediátricas. A utilização de protocolo de monitoração foi mais frequente em unidades de terapia intensiva com fisioterapeuta exclusivo e com tempo de atuação 24 horas/dia.

## INTRODUCTION

Cuffed or uncuffed tracheal intubation is a common procedure in emergency units, intensive care units (ICU) and surgical centers. The use of uncuffed tubes in children under 8 years of age is a routine practice in tracheal intubation considering the peculiarities of the laryngeal anatomy of children, such as the narrower subglottic region, the more cephalic position and more elliptical shape of the cricoid ring^(1)^ and the risk of injury to the airway mucosa.^(2)^

The use of cuffed tracheal tubes affords better sealing of the trachea, which consequently decreases the risk of bronchoaspiration,^(3)^ favors control of the pressure exerted on the tracheal mucosa via cuff pressure monitoring^(4)^ and reduces the risk of stridor after extubation as long as the cuff pressure is within the recommended safety limits.^(5,6)^ In addition to these factors, the use of cuffed tracheal tubes reduces the need to replace the tube due to air leakage, affords more reliable measurements of lung capacities and volumes, optimizes the use of capnography and does not cause increased morbidity in children with prolonged use of mechanical ventilation (MV).^(7,8)^

Studies^(9,10)^ in adults recommend a safe cuff pressure of between 20 and 30cmH_2_O; in pediatrics, the maximum suggested pressure is 20cmH_2_O.^(11,12)^ It is important to note that hyperinflation of the cuff and compression of the tracheal mucosa may lead to ischemia, fibrosis and subglottic stenosis, especially in the pediatric population.^(13)^

Recent improvements in cuffed tracheal tube design and components have provided greater safety in the pediatric age group. However, it is essential to monitor the intracuff pressure regardless of the patient’s age,^(7,14)^ which is a challenge in routine clinical practice.

There are limitations regarding the routine use of cuffed tracheal tubes in pediatric patients,^(15,16)^ especially in the neonates.^(12)^ There is also a lack of studies on the care of these tubes in both pediatrics^(5,17)^ and neonatology.^(17-19)^

In Brazil, there are no studies describing the use of cuffed tracheal tubes or the care of these devices in pediatrics and neonatology. The objective of the present study was to identify neonatal, pediatric and mixed (neonatal and pediatric) ICU in Brazil that use cuffed tracheal tubes in clinical practice and to describe the characteristics related to the use of protocols and monitoring.

## METHODS

A cross-sectional analytical survey was conducted by applying an electronic questionnaire with support from the *Rede de Cooperação em Pesquisa Clínica da Associação de Medicina Intensiva Brasileira* (AMIBnet/AMIB.) The study was approved by the Research Ethics Committee of *Universidade Federal de Uberlândia*, Uberlândia, Minas Gerais, Brazil (CEP: 1,301,015).

Through the Ministry of Health’s National Registry of Healthcare Facilities (*Cadastro Nacional de Estabelecimentos de Saúde* - CNES), information was obtained on 693 registered ICU, including 337 neonatal ICU 323 pediatric ICU and 33 mixed pediatric ICU. Mixed pediatric ICU were units that provided care to newborns, infants, children and adolescents.^(20)^

The minimum number of ICU that needed to participate in this study to ensure that the study sample was representative of ICU with these characteristics in Brazil and covered all states in the country was estimated. For the sample size calculation, a significance level of α = 0.05 and a test power of 1-β = 0.95 were considered, and the minimum sample size required was 82 ICU.

Subsequently, the name of the coordinator or intensivist (physician, physical therapist or nurse) responsible for the unit was collected, and he or she was contacted by telephone and/or e-mail. Telephone/e-mail contact was made with 298 ICU coordinators or supervisors, and an invitation letter was sent to each of them by e-mail containing an explanation of the study, a declaration of participation in the study and a link to access the questionnaire. The questionnaire was then sent to 298 ICU authorized by a coordinator or supervisor. Only one professional representing each unit was invited to answer the questionnaire on behalf of the multidisciplinary team working in the unit. Units whose coordinator/supervisor signed and forwarded the declaration of participation/consent participated in the study.

The electronic questionnaire was developed by the investigators using the Google Forms survey tool. It contained 16 questions, of which 8 were open and 8 were closed; the questions were related to the use of cuffed endotracheal tubes in the ICU, the use of cuff pressure monitoring protocols, causes of extubation failure, the presence of a physical therapist and the amount of daily care time provided by the physical therapist in the ICU (Appendix 1).

Statistical analysis was performed using the free software R^(21)^ and the chi-square test for multiple proportions was used for categorical variables. The results are presented as the frequency, percentage, and mean ± standard deviation. A significance level of 0.05 was adopted in the analyses.

## RESULTS

**Figure 1:**
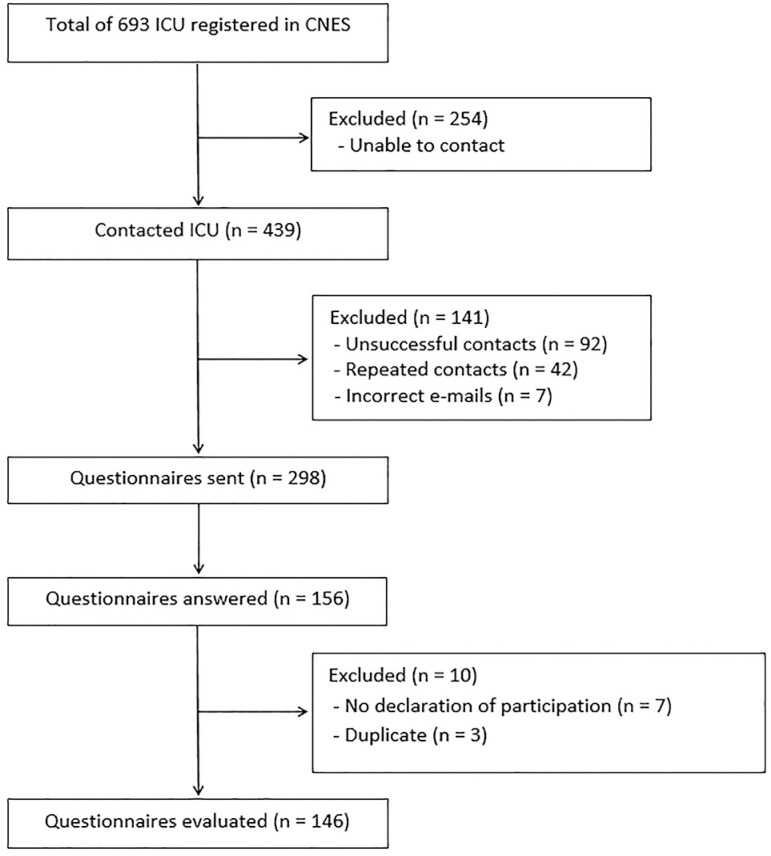
Flowchart of intensive care unit selection.

Of the 298 questionnaires sent, 156 (52.3%) were answered. Of these, 146 (48.9%) were analyzed, and 10 were excluded (3 because they were duplicates and 7 because the participation form was not included) (Figure 1). Of the 146 participating units, 72 (49.3%) were neonatal ICU, 52 (35.6%) were pediatric ICU, and 22 (15.1%) were mixed pediatric ICU. The median (minimum - maximum) admission age of the patients from the three ICU types were as follows: maximum age in neonatal ICU, 28 (14 - 540) days; minimum age in pediatric ICU, 30 (0 - 60) days; maximum age in pediatric ICU, 15 (12 - 21) years; maximum age of newborns in mixed pediatric ICU, 28 (28 - 60) days; minimum age of children in mixed pediatric ICU, 30 (0 - 30) days; maximum age of children in mixed pediatric ICU, 14 (12 - 18) years. In the neonatal and mixed pediatric ICU, the proportions of newborns weighing less than 2,500g over the last year were 69% and 64%, respectively. The proportions of newborns weighing less than 1,500g in these units were 31% and 36%, respectively. In the pediatric ICU, the proportions of patients by age group at admission over the last year were as follows: 85% were between 1 month and 5 years old; 11% were between 6 and 10 years old; and 4% were above 11 years of age. In the mixed pediatric ICU, 77% of the admitted children were between 1 month and 5 years of age; 14% were between 6 and 10 years of age; and 9% were above 11 years of age.

ICU - intensive care unit; CNES - Cadastro Nacional dos Estabelecimentos de Saúde.

Table 1 shows the results related to the use of cuffed tracheal tubes and the availability of the physical therapy service in the ICU. The use of cuffed tracheal tubes was predominant, occurring in 53.4% of the participating units (78/146). Among the units that used cuffed tracheal tubes, there was a significantly higher use in pediatric ICU (52/78) than in mixed pediatric ICU (21/78) and neonatal ICU (5/78) (Table 2).

**Table 1:** Data related to the participating intensive care units (n = 146)

Variables	
Profession of the respondent	
Physician	92 (63.0)
Physical therapist	49 (33.6)
Nurse	5 (3.4)
Use of cuffed tracheal tubes	
Yes	68 (46.6)
No	68 (46.6)
Rarely	10 (6.8)
Use of a cuff pressure monitoring protocol (n = 78)*	
Yes	45 (57.7)
No	33 (42.3)

Results expressed as n (%).

* The number in parentheses reflects the number of respondents for each item.

**Table 2:** Comparison among intensive care units types regarding the use or nonuse of cuffed tubes

ICU type	Uses cuff		Total	Incidence	RR	95%CI (RR)
	Yes	No				
Pediatric/mixed pediatric	73	1	74	98.65	14.21	6.10 - 33.10
Neonatal	5	67	72	6.94		
Total	78	68	146	53.42		

ICU - intensive care unit; RR - relative risk; 95%CI - 95% confidence interval. Results expressed as n. If the relative risk is > 1, the probability of using cuffed tubes is higher for patients in pediatric and mixed pediatric intensive care units than for those in neonatal intensive care units.

Regarding the application of a cuff pressure monitoring protocol, more than half of the ICU (45/78) used one, and the mean cuff pressure used was 23.6 ± 6.19cmH_2_O. Table 3 shows significantly greater use than nonuse of cuff monitoring protocols in pediatric ICU (32/45). There was no significant difference in the use or nonuse of a cuff monitoring protocol in the mixed pediatric ICU (10/21 *versus* 11/21) or the neonatal ICU (3/5 *versus* 2/5).

**Table 3:** Comparison among intensive care unit types, physical therapy care time per day and use or nonuse of a cuff pressure monitoring protocol

	Cuff pressure monitoring protocol	
	Yes	No
ICU type		
Neonatal	3^aB^	2^aB^
Pediatric	32^aA^	20^bA^
Mixed pediatric	10^aB^	11^aA^
Has a physical therapy service		
Yes, and it is exclusive to the ICU	38^aA^	23^bA^
Yes, and it is not exclusive to the ICU	7^aB^	9^aB^
Physical therapy hours/day		
24 hours/day	25^aA^	14^bA^
18 hours/day	10^aB^	3^bB^
< 18 hours/day	10^aB^	14^aA^

ICU - intensive care unit. Results expressed as n. Values in the columns followed by the same superscript uppercase letter do not differ significantly according to the chi-square test for multiple proportions considering a significance level of 5% (p < 0.05). Values in the rows followed by the same superscript lowercase letter do not differ significantly according to the chi-square test for multiple proportions considering a significance level of 5% (p < 0.05).

In the ICU with a physical therapy service exclusive to the unit, there was predominance of the use of a cuff monitoring protocol (38/61). In addition, at these units, the use of the protocol was significantly higher (38/45) when compared to the units that also applied a cuff monitoring protocol but did not have a Physical Therapy Service exclusive to the unit (7/45).

Units with a physical therapist present 24 hours a day had significantly higher rates of use of a cuff monitoring protocol (25/45) than units with a physical therapist present for 18 hours a day (10/45) or for less than 18 hours a day (10/45). Use of a cuff monitoring protocol was significantly higher than nonuse in both the ICU with a physical therapist present for 24 hours a day (25/39 *versus* 14/39) and those with a physical therapist present for 18 hours a day (10/13 *versus* 3/13).

The causes of extubation failure related to the use of a cuff in pediatric ICU were analyzed as only a small portion of the neonatal ICU (5/72) used cuffed tracheal tubes, and in the mixed pediatric ICU, cuff use was not inquired about separately for neonates and children. All observed causes of extubation failure were related to the use of cuffed tracheal tubes, and the most frequently reported cause was upper airway obstruction. The relationship between the causes of extubation failure and the use of a cuff monitoring protocol is shown in table 4.

**Table 4:** Comparison of causes of extubation failure, cuff use and use of a cuff pressure monitoring protocol in pediatric intensive care units

Pediatric ICU (n = 52)	Cuff use		Cuff pressure monitoring	
	Yes	No	Yes	No
Causes of extubation failure*				
Increased respiratory distress	23	0	14	9
Younger age	8	0	5	3
Longer MV duration	21	0	10	11
Longer sedation time	18	0	10	8
Upper airway obstruction	31	0	20	11
Chronic respiratory disease	15	0	9	6
Neurological disease/NMD	27	0	16	11
Congenital heart disease	4	0	2	2
Genetic syndrome	8	0	6	2
Malnutrition	9	0	3	6

ICU - intensive care unit; MV - mechanical ventilation; NMD - neuromuscular disease. Results expressed as n.

* Respondents were able to select more than one answer option.

## DISCUSSION

This was the first study in Brazil that evaluated the practice of using cuffed tracheal tubes in neonatal, pediatric and mixed ICU. Frequent use of cuffed tracheal tubes was observed in pediatric and mixed pediatric ICU. The application of a cuff pressure monitoring protocol occurred mainly in pediatric ICU, in ICU that had an exclusive physical therapy service and in those with a physical therapist present 24 hours a day.

A study conducted in the United Kingdom in 2008 showed that the routine use of cuffed tracheal tubes occurred in only 5% of the evaluated pediatric ICU and in 7% of neonates during anesthesia.^(2)^ In 2015, a survey conducted in Finland, Sweden, Norway and Denmark showed that cuffed tracheal tubes were used in 50% of pediatric ICU, and they were used more frequently in units managed by anesthesiologists.^(22)^ Another survey conducted in Australia and New Zealand in 2015 showed that 100% of the pediatric ICU and 33.3% of the neonatal ICU evaluated used cuffed tracheal tubes, and the most frequent use occurred in neonates over 3kg and infants under three months.^(23)^ However, the use of cuffed tracheal tubes in neonates under 3kg was infrequent. Our findings were similar, albeit we found an even lower frequency of cuff use in neonatal ICU (6.9%).

Although the use of cuffed tracheal tubes varies in different regions, studies^(7,24,25)^ have demonstrated that they are safe to use in children and are associated with a reduced need for tube replacement and no increase in the risk of after extubation stridor compared to the use of uncuffed tracheal tubes.

In this study, a lower frequency of cuffed tracheal tube use was observed in neonatal ICU, which demonstrates their restricted use in the neonatal population. This finding may be related to the peculiarities of this age group and the need for prolonged intubation, which injures the mucosa when cuffs are used, especially in cases in which there is no monitoring of the pressure exerted on the airway.^(10)^

Despite these considerations, there are advantages to using cuffed tracheal tubes in the neonatal population, especially for admitted neonatal postoperative patients.^(23)^ However, it is still not a common practice, especially in preterm infants and those under 3,000g, due to the lower availability of tracheal tubes with an adequate cuff size for this population. It is known that ultra-thin polyurethane tubes can be safely used in neonates under 3,000g because these tubes were specially developed for the pediatric population and have smaller cuffs, which facilitates their placement below the subglottic region rather than at the level of the cricoid cartilage.^(23)^

Although the use of cuffed tracheal tubes has increased in clinical practice, there is still no recommendation regarding cuff pressure monitoring. In this study, a protocol for cuff pressure monitoring was mainly used in pediatric ICU. In the United Kingdom, a study showed that 45% of the evaluated pediatric ICU did not monitor cuff pressure routinely,^(2)^ and the same was true in France, where cuff pressure monitoring in pediatric ICU was also infrequent.^(15)^ A survey conducted in 2016 with members of the Society for Pediatric Anesthesia (SPA) in the United States, Canada, Europe and other countries showed that more than 60% of the respondents did not monitor cuff pressure during anesthesia.^(26)^ To date, there are no scientific studies on cuff pressure monitoring and the use of cuff monitoring protocols in neonatal ICU.

The use of a cuff pressure monitoring protocol occurred mainly in ICU that had a physical therapy service exclusive to the unit and in those with a physical therapist present 24 hours a day. Practices related to cuff pressure monitoring care and the characteristics of the professionals involved in this process are rarely described in the literature. In North America, cuff care is managed by respiratory technicians, while in Brazil, there is no standardization of the professional responsible for this monitoring.^(27)^

In clinical practice, care for the tracheal cuff often occurs through interprofessional collaboration among nurses, medical staff^(27)^ and physical therapists. Among other functions, the ICU physical therapist, along with the multiprofessional team, addresses the management of invasive and noninvasive MV, protocols related to MV and care and monitoring of the tracheal cuff.^(28)^

Surveys^(29-31)^ conducted previously showed that ICU that have a physical therapist exclusive to the unit, especially those with a physical therapist present 24 hours a day, 7 days a week, use MV-related protocols more frequently. Such protocols are useful for the standardization of care, procedures and clinical parameters, and they contribute to the involvement of the multiprofessional team in actions that can improve quality of care and avoid unnecessary variations in clinical practice.^(29.31)^

Another result observed in this study was the higher frequency of use of a maximum cuff pressure above 20cmH_2_O in pediatric patients in Brazil. In the United Kingdom,^(2)^ the maximum pressure used in pediatric ICU ranges from 15 - 20cmH_2_O, and in Australia and New Zealand, it is 20cmH_2_O.^(12)^ It is known that the tracheal mucosal perfusion pressure in adults is approximately 30cmH_2_O.^(19)^ Compression of the tracheal mucosa associated with elevated cuff pressure can lead to ischemia, fibrosis and subglottic stenosis.^(13)^

Studies^(32.33)^ on adult patients have shown that a cuff pressure above 20cmH_2_O is desirable for preventing the aspiration of contaminated supraglottic secretions and, consequently, preventing ventilator-associated pneumonia (VAP).^(34)^ The frequency of lower airway microaspiration is higher in children on MV due to a longer ventilation duration, the absence of a tracheal cuff, younger chronological age, bronchopulmonary dysplasia and the presence of tracheostomy.^(3)^ No studies were found describing the minimum pressure required to avoid the aspiration of supraglottic secretions into the lower airway in children. However, it is known that cuffed ultrathin polyurethane tracheal tubes require cuff pressures of less than 15cmH_2_O to effectively seal the trachea.^(35)^

The stability of the cuff pressure depends on numerous factors, such as tracheal and cuff compliance,^(36)^ the position of the patient and cuff,^(37)^ the cuff filling volume^(9)^ and body temperature.^(38)^ These factors are constantly changing during the ICU stay, and thus, cuff pressure monitoring and adjustment are continuously and routinely required.^(39)^

Regarding the causes of extubation failure in pediatric ICU, upper airway obstruction was the most frequent cause related to the use of cuffed tracheal tubes, despite the application of an internal pressure monitoring protocol. Previous studies^(40,41)^ showed that upper airway obstruction was the most frequent cause of extubation failure in children. Other studies^(5,42)^ have shown that there was no association between the outcome of extubation and the use of cuffed or uncuffed tracheal tubes.

This study had some limitations because its nature as a voluntary survey may have led to lower participant adherence. Some ICU were not included because the study participation/consent form was not received. This study included all the states and regions of Brazil. The sample size of participating ICU was above the minimum sample size required (82 units) to be representative of the total number of ICU in Brazil.

This is the first study to evaluate the use of cuffed tracheal tubes and the care related to them in neonatal, pediatric and mixed pediatric ICU in Brazil. The strict monitoring of cuff pressure should be desired as a measure of the quality of care for critically ill neonatal and pediatric patients.^(27)^ This study was important for analyzing and understanding the relationship between the length of time the physical therapist works in the ICU and its correlation with the application of protocols for adequate airway care, specifically monitoring of tracheal tube cuff pressure.

## CONCLUSION

This study showed that in pediatric intensive care units in Brazil, the frequencies of application and monitoring of cuffed tracheal tubes are higher than in neonatal and mixed intensive care units and that the application of a cuff monitoring protocol was more common in intensive care units that had a physical therapist who was exclusive to the unit and was present 24 hours a day. Prospective and multicenter studies are needed to evaluate the safety and effectiveness of the use of cuffed tracheal tubes in the neonatal and pediatric population and to examine the development of standardized care protocols.

## Appendix 1 Questionnaire

**Table:**

**Questionnaire on the use of cuffed tracheal tubes in Brazilian pediatric and neonatal ICU**

**Institution and ICU coordinator identification:**
Important: The information below will be kept confidential and anonymous. It will be used only for internal research control.

**1. Name of institution:**
**2. Name of coordinator responsible for the ICU:**
**3. City of the institution/ICU:**
**4. State:**

**Respondent identification:**
Important: The information below will be kept confidential and anonymous. It will be used only for internal research control.

**5. Full name:**

**6. Telephone: (area code) fixed and mobile numbers:**

**7. Contact e-mail:**

**8. Occupation:**
( ) Physician.
( ) Physical therapist.
( ) Nurse.

**ICU characteristics**

**9. Does the ICU have a physical therapy service?**
( ) Yes, and the physical therapist is EXCLUSIVE to the unit.
( ) Yes, but the physical therapist is NOT EXCLUSIVE to the unit.
( ) No.
( ) Don't know.

**Data on the physical therapy service**

**10. What is the total time the physical therapy service is available in a 24 h period?**
( ) 24 hours/day.
( ) 18 hours/day.
( ) 12 hours/day.
( ) Other ______________________________________________________________

**ICU profile**

**11. What type of ICU do you work at?**
( ) Neonatology.
( ) Pediatrics.
( ) Mixed (neonatology and pediatrics).

**Data on neonatal patients (in neonatal and mixed ICU)**

**12. What are the main causes of extubation failure in neonatal patients at your ICU?** **Note: Select the 2 to 3 most frequent causes.**
( ) Apnea.
( ) Increased respiratory distress.
( ) Clinical deterioration (hemodynamic, infectious and neurological).
( ) Accidental extubation.
( ) Upper airway obstruction.
( ) Longer mechanical ventilation duration.
( ) Lower birthweight (< 1,000g).
( ) Low Apgar scores.
( ) Younger age at extubation.

**Data on pediatric patients (in pediatric and mixed ICU)**

**13. What are the main causes of extubation failure in pediatric patients at your ICU? Note: Select the 2 to 3 most frequent causes.**
( ) Increased respiratory distress
( ) Younger age (infants < 24 months)
( ) Longer mechanical ventilation duration
( ) Longer sedation time
( ) Upper airway obstruction
( ) Chronic respiratory disease
( ) Neurological or neuromuscular disease
( ) Congenital heart disease
( ) Genetic syndrome
( ) Malnutrition

**Cuffed tracheal tube use**

**14. Does the service use cuffed tracheal tubes?**
( ) Yes, when necessary or indicated
( ) No
( ) Rarely

**15. Is there a cuff pressure monitoring protocol?**
( ) Yes
( ) No

**16. What is the maximum pressure value established?**
